# Thermal Transport in 2D Materials

**DOI:** 10.3390/nano13010117

**Published:** 2022-12-26

**Authors:** Mohammad Hassan Kalantari, Xian Zhang

**Affiliations:** Department of Mechanical Engineering, School of Engineering and Science, Stevens Institute of Technology, Hoboken, NJ 07030, USA

**Keywords:** 2D materials, thermal conductivity, simulations, experimental measurements

## Abstract

In recent decades, two-dimensional materials (2D) such as graphene, black and blue phosphorenes, transition metal dichalcogenides (e.g., WS_2_ and MoS_2_), and h-BN have received illustrious consideration due to their promising properties. Increasingly, nanomaterial thermal properties have become a topic of research. Since nanodevices have to constantly be further miniaturized, thermal dissipation at the nanoscale has become one of the key issues in the nanotechnology field. Different techniques have been developed to measure the thermal conductivity of nanomaterials. A brief review of 2D material developments, thermal conductivity concepts, simulation methods, and recent research in heat conduction measurements is presented. Finally, recent research progress is summarized in this article.

## 1. Introduction

Two-dimensional materials are characterized by excellent structural, mechanical, and physical properties, making them suitable for basic science and engineering applications because of their superb properties [1]. Throughout recent years, 2D nanomaterials have been the subject of extensive studies, resulting in massive interest in their applications in novel nanodevices with unique functions. A growing interest in understanding the thermal properties of 2D materials has been observed over the last few years. In addition, in nanomaterials, thermal transport has revealed many unique phenomena, which, when understood, will open up new possibilities for the development of new nanotechnologies in thermal management. New technologies have become increasingly dependent on thermal conductivity as an essential parameter. Many benefits can be derived from nanoelectronic devices using 2D materials and they may potentially extend electronics into new fields of application.

A temperature increase occurs when advanced materials are used in some electronic applications. Increased thermal conductivity allows heat to diffuse faster and prevents large overheating, which can result in premature degradation. The majority of these unique phenomena are due to nanomaterials’ notable properties. In fabrication and application, chemical functionalization, strain, and structural interruptions can alter their atomic structures, affecting their properties. Research on the micro/nano components of two-dimensional material has recently focused on their electrical, mechanical, and optical properties. It should be noted that for any micro/nano component, whether it is an electronic component or an optoelectronic component, the heat dissipation problem determines the device’s performance and stability. High-density components will generate a lot of heat during high-speed operation. If the heat cannot be eliminated in time, it will cause the components to be too high in local and performance degradation, or even burnout. How to conduct immense heat away so that the components work in a relatively low-temperature environment becomes a common issue in the modern semiconductor industry [2,3,4,5,6].

Most microelectronic devices are combined with semiconductors and metals so the contact interface between semiconductors and metals can be seen everywhere. Microelectronic devices’ heat dissipation problem involves the following physical issues: (1) How does heat transfer in micro/nano-scale materials? (2) How does heat pass through various interfaces? The most dominant heat carriers in semiconductor materials are phonons, which are found in micro/nano electronic devices. Consequently, the following questions arise: (1) How do phonons travel in micro/nano-scale semiconductor materials? (2) How do phonons pass through various interfaces? The study of these two problems enables us to solve heat dissipation problems related to micro/nano devices and thermal conductivity control.

An electronic device can generate heat in many different ways, such as by Joule heating, solar flux, or exothermic reactions. High-power density electronics such as integrated circuits, supercapacitors, LEDs, and lasers are notorious for localized Joule heating. Nanoscale devices have a higher power density, but a reduced amount of heat can be extracted as their dimensions decrease. Nanostructured solar cells and concentrated solar cells share a similar challenge of reducing efficiency with increased temperatures. For a final example, batteries can experience exothermic reactions and Joule heating, which may cause unwanted chemical reactions and device failure. Materials that must minimize heat transfer are at the other extreme. To reduce heat transfer across each leg, thermoelectric devices require materials with a low thermal conductivity. It creates a design conflict when thermoelectric materials must also be good electrical conductors. To prevent heat from reaching critical parts, thermal insulation is designed.

Advances in the electronics industry have fueled an enormous demand for pioneering thermal management strategies to enhance the performance and reliability of devices by controlling energy dissipation generated in the devices. In nanoelectronics, where heat dissipation is a vital factor in the performance of high-density nanoscale circuits, or in thermoelectric materials, where low thermal conductivity is desired, controlling heat diffusion by controlling the phononic properties of fundamental components is a major interest. For instance, among the promising candidates for field effect transistor applications with a high on/off ratio and high mobility, single-layer MoS_2_ is a semiconductor with a large bandgap of ~1.1–2 eV [7,8,9]. Low-temperature carrier mobility can be significantly enhanced by improving sample quality and using appropriate electrode materials [10]. Following these recent breakthroughs, it is highly expected that 2D materials will be used in integrated circuits (ICs) in the near future. Another example is stanene, a single-layer buckled honeycomb structure of tin atoms that exhibits near-room temperature quantum anomalous Hall effects [11] and ultra-low thermal conductivity [12], which makes it ideal for thermoelectric applications. This article discusses in detail the thermal conductivity of 2D materials.

## 2. Two-Dimensional (2D) Materials

Understanding material systems is at the core of the technology. Each application requires specific material properties. For example, circuits are built with copper because of their electrical conductivity, skyscrapers are constructed with concrete because of their compressive strength, and car tires are constructed with vulcanized rubber, which is pliable and durable. Technology can advance further as we gain a deeper understanding of a material’s properties. Nanomaterials refer to materials with a dimension of at least one nanometer in size. Qualitative changes in physicochemical properties and reactivity are related to the number of atoms or molecules determining the material in this scale. To illustrate, the surface plasmon resonance of metal nanoparticles and quantum confinement of semiconductor particles can be observed as size-effect properties. Recent years have seen an increased interest in two-dimensional (2D) nanomaterials due to their unique properties. Furthermore, two-dimensional nanomaterials bridge the gap between one-dimensional (1D) nanomaterials and three-dimensional (3D) bulk materials, raising new fundamental problems related to low-dimensional materials that can lead to a host of new applications. As these nanodevices become more widely available, the need to understand their thermal properties has increased. Two-dimensional nanomaterials are briefly introduced in this section.

Monolayer graphene flakes were isolated from bulk graphite by mechanical exfoliation, launching the field of two-dimensional (2D) materials [13]. There have also been numerous discoveries of 2D materials since then, including transition metal dichalcogenides (TMDs, e.g., MoS_2_), hexagonal boron nitride (h-BN), and black phosphorus (BP) (or phosphorene). There is a wide range of physical properties available within the 2D materials family, from conducting graphene to semiconducting MoS_2_ to insulating h-BN. As an added advantage, 2D crystal structures exhibit superior mechanical properties, exhibiting a high in-plane stiffness and strength, as well as an extremely low flexural rigidity. Together, the 2D materials have a wide range of potential applications [14,15].

Van der Waals forces or weak covalent bonds that hold together material layers can be mechanically or chemically exfoliated down to an in-plane, covalently bonded single layer. A 2D materials history dates back to the 1960s. As early as 1980, graphene, a one-atom thick graphite layer, was isolated and studied extensively as a monolayer. Novoselov and Geim introduced 2D materials by studying graphene under electric and magnetic fields [16]. Because of the high quality of the crystals and their ease of obtaining them, many researchers have developed more complicated 2D electron gas materials for graphene. A number of graphene effects were determined as a result. It has also been shown that other layered materials are known to mechanically exfoliate. Over a thousand materials have been identified by 2020 [17]. As layers often significantly influence the electrical, optical, and thermal properties of materials, this large number of materials can enable a plethora of novel physics. For example, by changing the gap energy, MoS_2_ transitions from an indirect to a direct bandgap as the monolayer limit is reached.

**Figure 1:**
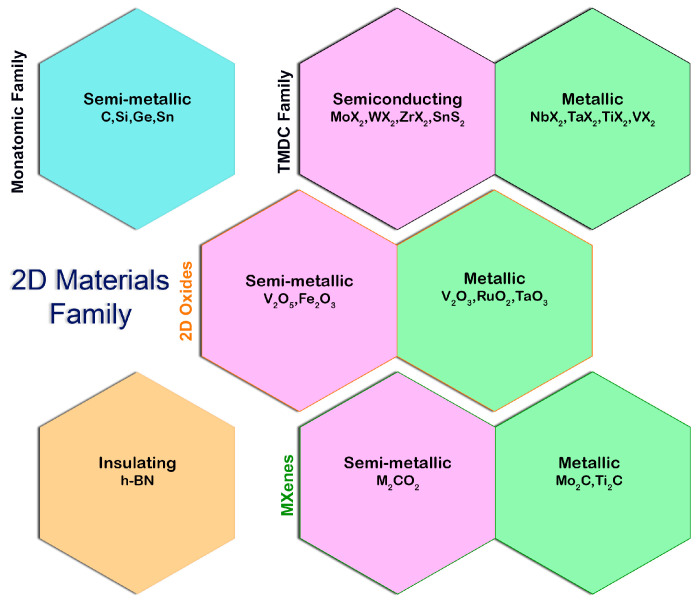
2D materials family [18]. Reprinted with permission from ref. [18]. Springer Nature and Copyright Clearance Center.

Material properties are often thought to be determined solely by their material composition. Electricity is conducted by metals because they contain metallic bonds between their atoms, allowing electrons to drift freely throughout the material. The strength of concrete comes from the cement that rigidly locks incompressible sand and gravel together. Vulcanized rubber is made of flexible polymer chains that are firmly linked together, making it both pliable and durable. The size of a material, however, can influence its behavior. Materials with nanoscale dimensions (i.e., whose sizes can be expressed in nanometers) are particularly susceptible to this. The nanoscale can affect electrical conductivity, chemical reactivity, mechanical properties, and even how a material interacts with light. A fascinating and unexpected new property of nanomaterials is being revealed as we become more adept at creating and studying nanomaterials. With this advancement, future technologies that rely on both bulk properties and material size have opened up entirely new opportunities. A new era of nanotechnology has begun. Periodic tables of 2D materials are currently being worked on and may offer a new form of chemistry using layers rather than atoms (Figure 1). In this article, the thermal properties of 2D materials are discussed, following many excellent literature reviews on the subject.

**Figure 2:**
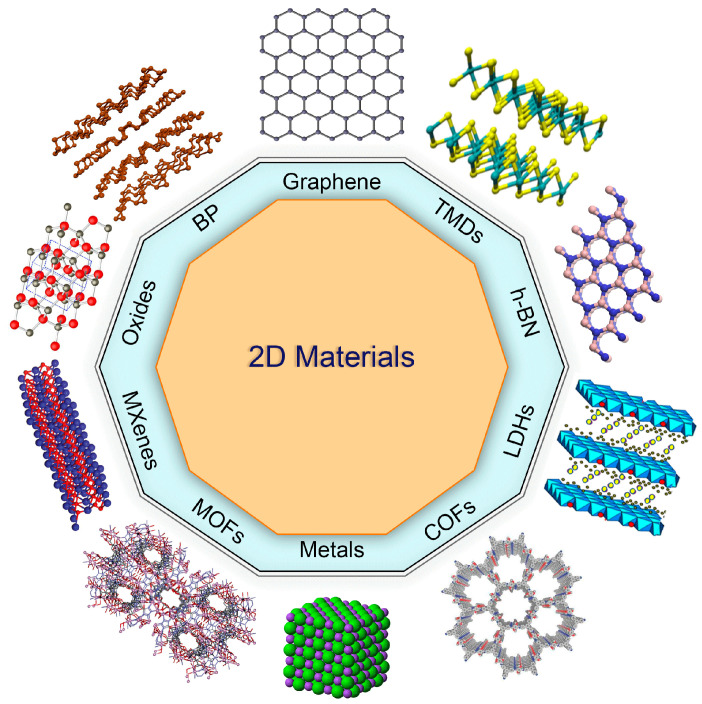
Some of the introduced 2D materials.

The first novel 2D material introduced was graphene in 2004. Since then, many other 2D materials have been proposed [17] with an extensive range of properties. In this section, several of the researchers’ materials of interest are presented briefly. The two types of 2D materials are single-element 2D materials (such as graphene, black phosphorus (BP), silicene, germanene, etc.) and compound 2D materials (such as TMDs, h-BN, TMCs, III–V group elements, compound semiconductors, etc.). Figure 2 below illustrates some of these types. 

Graphene is a semimetal that consists of a covalently bonded hexagonal lattice of carbon atoms. In most cases, it is only one-atom thick (about 0.14 nm). The distinctive band structure of graphene enables electrons to move rapidly at speeds close to 1/300 the speed of light, resulting in its excellent thermal conductivity and high tensile strength. A single monolayer of graphene could support the weight of an entire football [19]. 

Hexagonal boron nitride (h-BN) is an isomorph of graphene (behaves similarly in terms of its crystallographic properties), except instead of carbon, boron and nitrogen atoms make up the structure. It is a wide-bandgap insulator, in contrast to graphene.

TMDCs are transition metal dichalcogenides with the chemical formula MX_2_, where M is a transition metal (such as tungsten (W) or molybdenum (Mo)), and X represents a chalcogen (like selenium (Se), sulfur (S), or tellurium (Te)). A TMDC is made up of a metal layer sandwiched between two chalcogenide layers, with each layer being three atoms thick. The crystal structure of TDMCs can vary. A 2H-phase with trigonal symmetry is the most common, resulting in semiconducting properties like MoS_2_, WS_2_, and MoSe_2_. The bulk form of these semiconductors has an indirect bandgap. It is interesting for optoelectronics to use monolayers because their bandgap becomes direct and visible. The metallic 1T phase, the most stable polymorph of WTe_2_, is another example of such structures.

A single layer of black phosphorus (BP) is called phosphorene, which is a stable allotrope of elemental phosphorus. This semiconductor has a puckered honeycomb structure with a direct bandgap. Layers can be stacked on top of each other to tune the bandgap throughout the visible region. Due to this, these materials are appropriate for transistors and optoelectronic devices. The corrugated structure of phosphorene causes its properties to vary noticeably depending on its measurement direction.

MXenes are monolayers of tin (stanene), germanium (germanene), and silicon (silicene). Similar 2D materials have also been developed, such as antimony [20] and bismuth [21]. Bismuth is found to have the potential for magneto-electronic applications [22].

## 3. Thermal Conductivity

A greater understanding of the thermal properties of 2D nanomaterials is required due to their rapid development. The main factors contributing to this demand are as follows. First, electronic devices are subjected to ever-increasing thermal loads due to continuous miniaturization and component density increases. Electronic device components are getting smaller and smaller every year, according to Moore’s law. One of the crucial components of electronics is the field-effect transistor, which has now reached a channel length of 100 nm, and a 50 nm channel length is on the horizon. Thermal design has become an essential part of electronic device development at the nanoscale, as controlling heat is critical for reliability and performance.

Furthermore, at the nanoscale, electronic devices exhibit thermal transport characteristics that are dramatically different from those observed at the macroscale. The electrical−thermal design of the electronic device should also take these features into account. As the use and requirement of energy sources increases, practical and efficient solutions are required for energy generation, consumption, and recycling. Heat management can be improved by utilizing recently developed nanotechnologies and nanomaterials. In some cases, for efficient thermal dissipation, nanomaterials with high thermal conductivities are employed in nanoscale electronics. A low thermal conductivity is required to increase thermal conversion or preservation in other cases, such as in thermoelectric devices and thermal barrier coatings, nanomaterials, or nanoparticles.

Heat is carried primarily by phonons in the same way electricity is carried by electrons. A recent study demonstrated that phonons can carry and process information as well [23]. A variety of types of thermal logic devices have been developed theoretically and even experimentally, such as thermal rectifiers [24,25], thermal transistors [26], thermal logic gates [27], and thermal memory cells [28]. In a similar manner to electronic circuits, thermal circuits can be fabricated using these basic thermal components.

Two-dimensional nanomaterials have different thermal properties than bulk materials due to their atomic structures. For example, graphene as a 2D material has a thermal conductivity as high as ~2000 W/m·K [29], or even higher [30]. This is comparable to the highest thermal conductivity material found in nature, which is diamond. As a result, high-power electronics could potentially benefit from its use in thermal management. h-BN also has high mechanical strength and good thermal properties. High-quality bulk h-BN samples could reach a thermal conductivity of ~390 W/m·K, indicating its potential as a current generation dielectric material [31]. In a study by Joe et al., the thermal conductivity of an 11-layer sample was found to reach about ~360 W/m·K [32]. For silicene, different MD simulations calculated thermal conductivities ranging from 5 to 50 W/m·K [33,34]. TMDs show different thermal conductivity. As an illustration, it has been estimated that MoS_2_ has a thermal conductivity of about 26 W/m·K, according to Wei et al. [35]. WS_2_ CVD-grown monolayer and bilayer thermal conductivity was determined by Peimyoo et al. For monolayer and bilayer WS_2_, the measured values are 32 and 53 W/m·K, respectively [36]. Theoretically, the thermal conductivity of phosphorene indicates low thermal conductivity. For instance, Qin et al. studied the simulated thermal conductivity along zigzag (ZZ) and armchair (AC) directions and found it to be 15.33 and 4.59 W/m·K, respectively [37]. However, during the manufacturing of 2D nanomaterials, structural defects such as voids, grain boundaries, and dislocations could be formed. A brief review of the thermal conductivity concept is provided in this section.

We review alternative approaches to determining temperatures and heat rates for a two-dimensional, steady-state conduction in the first step. A wide range of approaches are being used, which range from exact solutions that can be obtained for idealized conditions to approximate methods that vary in complexity and accuracy. In the following section, we consider some of the mathematical issues associated with obtaining an exact solution [38].

### 3.1. General Considerations and Solution Techniques

A heat flux equation, according to Fourier’s law, can be expressed as follows [38]:

(1) $$\frac{\partial }{\partial x}\left(k\frac{\partial T}{\partial x}\right)+\frac{\partial }{\partial y}\left(k\frac{\partial T}{\partial y}\right)+\frac{\partial }{\partial z}\left(k\frac{\partial T}{\partial z}\right)+\dot{q}=\rho c_{p}\frac{\partial T}{\partial t}$$

Here, $\dot{q}$ is the rate of energy generated per unit volume of the medium (W/m^3^) and *k* is the thermal conductivity (W/m⋅K). In Cartesian coordinates, Equation (1) is the general form of heat diffusion. There are two primary objectives that are usually attached to any conduction analysis. Known as the heat equation, it provides the basic tool for analyzing heat conduction. *T* (*x*, *y*, *z*) can be calculated as a function of time from its solution. For the present problem, the first objective is to detect the distribution of temperature in the medium and in order to do so, it is necessary to determine *T* (*x*, *y*). Solving the heat equation in the appropriate form is the key to obtaining this objective. It is found that in two-dimensional steady-state conditions with no generation and constant thermal conductivity, this form can be calculated from Equation (1) as follows:

(2) $$\frac{\partial^{2}T}{\partial x^{2}}+\frac{\partial^{2}T}{\partial y^{2}}=0$$

Equation (2) can be solved analytically by an exact mathematical solution. However, despite the fact that there are several techniques that can be used to solve these equations, the solutions usually involve complicated mathematical functions and series, and only a limited number of simple geometries and boundary conditions can be used. As a result of the dependent variable *T* being a continuous function of the independent variables (*x*, *y*), the solutions are valuable. Therefore, this solution can be used to calculate the temperature at any point within the medium.

A method of separation of variables is also used to compute an exact solution to Equation (2) in order to illustrate the nature and importance of analytical methods. For typical geometries that are usually existing in engineering practice, conduction shape factors and dimensionless conduction heat rates are sets of existing solutions. Graphical and numerical methods, on the other hand, can produce approximate results at discrete points, as opposed to analytical methods, which deliver exact results at any point. Although computer solutions based on numerical methods have replaced the graphical or flux-plotting methods, they can still be used to estimate temperature distribution quickly. Generally, it is used for two-dimensional problems with adiabatic and isothermal boundary conditions. A numerical method, on the other hand, can be used to obtain accurate results for complex, three-dimensional geometries involving an array of boundary conditions, in contrast to analytical or graphical approaches.

### 3.2. The Method of Separation of Variables

Two-dimensional conduction problems can be solved with the separation of variables method by applying the boundary conditions to a thin rectangular plate, a long rectangular rod, or any other shape that can be described by boundary conditions. By solving the heat equation, the temperature T corresponding value, heat flux, and heat flow lines can be determined, but this method is limited, complicated, and time-consuming [38]. For a variety of other geometries and boundary conditions, including cylindrical and spherical systems, exact solutions were obtained [39,40].

### 3.3. *The Conduction Shape Factor*

The process of finding an analytical solution to a two-dimensional or three-dimensional heat equation can be time-consuming and even impossible in some cases. This leads to the consideration of a different approach. For example, the heat diffusion equation can be quickly solved in many examples by employing existing solutions to it to solve two- or three-dimensional conduction problems. Shape factor *S* or steady-state dimensionless conduction heat rates $q_{ss}^{*}$ are used to present these solutions [38].

In some cases, it may be possible to provide accurate mathematical solutions to steady, two-dimensional conduction problems by using analytical methods, as outlined above. For a variety of simple geometry and boundary conditions, these solutions have been generated [41,42,43]. In spite of this, there are many two-dimensional problems that do not involve simple geometries and boundary conditions that would allow them to be solved with such solutions. A numerical approach such as finite-difference, finite-element, or boundary element may be the best choice in these cases in order to solve the problem.

Although the corresponding κ may differ significantly between 2D materials (such as graphene, molybdenum sulfide, and black phosphorus) and their bulk counterparts, taking advantage of these differences can lead to new possibilities in a variety of applications, such as thermal management and energy conversion. It is necessary to study the microscopic picture of two-dimensional materials in order to understand the heat transfer properties of these materials. This section will discuss some general fundamentals and concepts of phonon thermal transport before discussing details of 2D materials.

### 3.4. Thermal Transport at the Nanoscale

**Figure 3:**
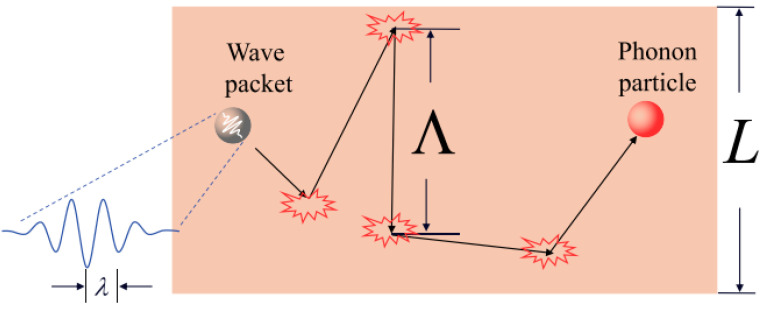
The schematic for the phonon particle picture [44]. Reprinted with permission from ref. [44]. Elsevier and Copyright Clearance Center.

A brief description of thermal transport at the microscale is necessary before discussing different effects in 2D materials. In order to transfer energy from one region of space to another region of space, the transportation or conduction of thermal energy requires the use of carriers, such as particles or waves. Except for alloys with extremely low electrical conductivity, metals conduct thermal energy mainly through electrons [44]. As shown in Figure 3, from a microscopic perspective, in dielectrics and semiconductors, it can be seen that heat is primarily carried by phonons or quantized vibrations of atoms in the lattice that can function as a particle to represent the phonon wave packets, according to the quantum state during their production. A phonon, when viewed from the angle of energy, will behave as a particle and collide with other phonon particles, as well as impurities and boundaries around it.

Firstly, before starting the detailed analysis, it is crucial that the length scales be clarified in advance. The figure shows a structure with a size of *L* (in 2D materials, *L* can be considered as the material’s thickness) and a wavelength of *λ* for phonon wave packets. It is noted that the phonon is considered to be a particle in the spherical regime (gray regime). Phonons collide with other phonons, impurities, and boundaries when they move within solids. A phonon mean free path *Λ* is a distance between two collisions, which is an incredibly significant concept in the field of thermal transport. At room temperature, the mean free path *Λ* typically ranges from nanometers to tens of micrometers. It can be much longer at low temperatures. Generally, transport properties are discussed primarily on length scales larger than phonon wavelengths *λ* and comparable to or smaller than mean free paths *Λ*. When size *L* exceeds the mean free path *Λ*, the size effect will not be taken into consideration, which echoes the bulk of classical Fourier’s law. A smaller size *L* than the mean free path *Λ* will result in phonons scattering on the boundaries before further phonon-to-phonon scattering. Due to these extra scatterings on the boundary, heat transfer will then be constrained by the boundaries. A primary cause of the reduction in thermal conductivity of 2D materials can be attributed to this phenomenon. It is known as the classical size effect when this type of effect occurs. In cases where the size *L* is smaller than the wavelength *λ*, we will encounter a quantum size effect as a result. A 2D material with a thickness of *L* usually exceeds the wavelength of the phonons unless the temperature is very low [44].

Materials, even crystals, do not have infinite thermal conductivity because phonons are scattered with one another. As result of this scattering, it is known as phonon−phonon scattering. High temperatures lead to stronger scattering and shorter mean free paths *Λ*. There is a great deal of complexity involved when it comes to phonon–phonon scattering, and it can also be very challenging to determine the phonon–phonon scattering time. As a result of the advancement of computation algorithms and the availability of more powerful computation capabilities, there have been noticeable advances in the calculation of the phonon–phonon scattering rate through the first principle. The following section provides an overview of some of these methods.

## 4. Simulation Methods

Advancement of computers and technology resulted in the development of atomistic models that have become so precise that they can typically be compared with experimental results. The use of atomic simulations has therefore become more common in nanomaterials research in the past few decades.

### 4.1. *Atomistic Simulations of Thermal Transport*

The thermal transport properties of a material can be predicted using atomic simulations by understanding its atomic structure and interatomic interactions. A variety of atomistic simulation approaches have been developed to study nanomaterial thermal transport properties.

To study the thermal transport properties of 2D materials, various theoretical methods have been introduced, including molecular dynamics simulations (MD), Boltzmann transport equations (BTE), and atomistic Green’s functions (AGF).

### 4.2. Introduction to Simulation Approaches

**Figure 4:**
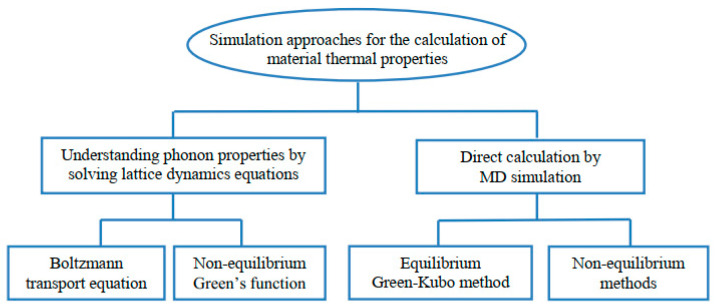
Materials thermal property classifications based on atomistic simulations [45]. Reprinted with permission from ref. [45]. Taylor & Francis Group LLC—Books.

In Figure 4, a variety of simulation techniques are presented for the study of material thermal properties [45]. The Boltzmann transport equation and the non-equilibrium Green’s function are examples of first-class approaches. In each of these methods, the thermal properties are predicted by solving the lattice dynamics equations based on understanding the fundamentals of phonon properties. Direct MD simulation is used in the second class for calculating thermal properties, such as the equilibrium Green−Kubo approach and the non-equilibrium MD method (also called the direct approach).

In the case of first-class methods, such as Monte Carlo (MC), during the calculation, phonon transport and scattering are taken into consideration. As a result, prior knowledge of phonon transport is needed. Obtaining such a requirement is easy if the material has a simple lattice structure. On the other hand, phonon transport is usually difficult to predict when there are structural disruptions, such as sharp interfaces. Contrary to the first-class approaches, the MD simulation in the second class uses Newton’s equations of motion in terms of time for a group of atoms interacting with each other via potential empirical functions. Furthermore, MD simulations are capable of modeling both small and large systems to calculate material thermal properties using phonon properties and solving lattice dynamics equation simulations, while a system’s size can be modeled. The following brief overview presents three of the more commonly used simulation methods for predicting the thermal conductivity of nanostructured materials.

### 4.3. A Mento Carlo Simulation Method

Boltzmann’s equation for the transport of phonons usually forms the basis of a theoretical analysis of phonon transport [46]. To solve this equation in a closed-form manner, many critical assumptions and simplifications must be made, which can cause huge deviations from experimental observations, especially for materials whose geometrical and lattice structure are relatively complicated and whose defects are multiple types. The MC approach was originally developed as a numerical solution to the Boltzmann equation in the context of electron transport [46]. The method has been widely applied since then to manage the transport problems of particles. In bulk materials [47], thin films [48,49], nanowires [50], and nanocomposites [51], the MC simulation has been successfully applied to determine thermal transport properties.

In the MC method, phonons are treated as random particles drifting in space when solving the transport problem. While MC simulations are typically used to predict thermal properties using lattice dynamics, i.e., phonons, this approach lacks the disadvantage that a numerical expression of the phonon dispersion is required to obtain reasonable phonon numbers and distributions in both the spatial and spectral spaces. A clear understanding of the scattering rate $\tau^{-1}\left(\omega \right)$ resulting from different scattering mechanisms should also be provided in order to address scattering events. For GE-based nanomaterials, these two requirements necessitate a lot of considerations, including strain, chemical functionalization, interface, and defects.

### 4.4. First Principles Method

Using the Boltzmann transport equation (BTE) in conjunction with the Schrödinger equation, first principles calculations can be performed on thermal transport. For the first principles calculation, no fitting parameters are required, as opposed to the traditional method of extracting phonon scattering times. The following steps are involved in the first principles of the thermal transport-based method. As a first step, first principles simulation refers to solving the Schrödinger equation numerically. Numerical computation is performed to calculate the atomic potential force constant. Based on these force constants, the anharmonic lattice dynamics will be used to extract the phonon dispersion relation and scattering rate. Then, the Boltzmann transport equation (BTE) can be linearized and solved numerically as well. As a result of this process, both the dispersion relation and the phonon scattering rate (or the relaxation time) for each phonon mode are calculated. Lastly, in order to calculate thermal conductivity, the lattice thermal connectivity can be extracted.

A wide range of 2D materials have been studied by using the first principles method since its development, including graphene [52,53], phosphorene [54,55], molybdenum disulfide (MoS_2_) [56,57], and silicene [34,58]. More detailed explanations of this method can be found in a number of outstanding review papers [59,60] or books [61].

### 4.5. Molecular Dynamics Simulations Method

There is also another widely accepted method for thermal transport in 2D materials known as Molecular Dynamics (MD), which relies on Newton’s law of motion as its physical foundation. Starting with the atomic potential between atoms, the process begins. As a result, the force acting on each atom can be calculated, as well as its velocity at any given moment. In the modern era of supercomputers, it is possible to determine the location of every atom at any time. Then, based on statistical mechanics principles, it is possible to study the expected macroscale properties.

To calculate the material’s thermal conductivity directly during MD simulation, non-equilibrium MD and equilibrium Green−Kubo approaches are most commonly used. A non-equilibrium MD approach is similar to the experimental measurement of thermal conductivity. As a result of this method, one region of the simulation cell is heated up, and another region that is situated at a distance is cooled down. As soon as the system reaches a stable state, it is possible to extract the temperature profile between the hot and cold regions, from which the temperature gradient between the two regions can be determined. It is a direct approach that can be easily implemented in MD simulation since it relies only on classical quantities such as force, velocity, and position to compute the temperature and heat flux. Furthermore, the calculation of the thermal properties does not require any significant assumptions to be made.

A note should be made here that the accuracy of classical MD calculations is highly dependent on the quality of the interatomic potentials. Recent studies have focused on constructing a reliable potential function from first principles calculations [62]. To obtain the thermal properties of 2D materials, equilibrium MD (EMD) and non-equilibrium MD (NEMD) simulations are modified. The Green−Kubo formula or the Einstein Relation ratio is used to calculate thermal conductivity in EMD [63,64]. The hot and cold reservoirs are connected to each side of a sample for NEMD simulations. To calculate thermal conductivity, the stationary-state heat flux and temperature are extracted after sufficient run-time in terms of Fourier’s law of heat conduction.

A major advantage of MD is that it is of atomic scale, yet it can be applied to large structures. Moreover, the computational cost can be significantly reduced when compared to the first principles approach. Therefore, the MD method is able to simulate systems that are several orders of magnitude larger than a first principle. The simulation can include millions, or even billions, of atoms. A 2D material can benefit from this since one direction is isolated. For example, recent research has modeled the thermal rectification device employing different graphene geometries [65]. This method can only be applied to systems with well-known potentials, such as carbon-based materials, due to the limited number of potentials identified and verified. Machine learning algorithms have become extremely useful methods of calculating atomic potentials over the past few decades [59]. We can expect more reliable potentials as these algorithms develop and computational power increases.

The simulation of nanoscale objects, such as GE, is usually carried out by either first principles simulations or molecular dynamics simulations (MD). MD simulations are a suitable alternative to first principles simulations because they allow the system size to get relatively large compared to first principles simulations. A study of nanomaterials’ thermal properties can thus be facilitated by this method.

### 4.6. Equilibrium Green−Kubo Approach

According to the equilibrium Green−Kubo approach, thermal conductivity is calculated by monitoring the dissipation time of these fluctuations. The Green−Kubo approach calculates the thermal conductivity of an isotropic material as follows [60]:

(3) $$\lambda =\frac{1}{3k_{B}VT^{2}}\overset{\infty }{\underset{0}{\int }}\langle \vec{J}\left(0\right)\vec{J}\left(t\right)\rangle dt$$

where $\langle \vec{J}\left(0\right)\vec{J}\left(t\right)\rangle$ is the autocorrelation function for heat flux and the angular brackets demonstrate ensemble averages. Here, *T* is the temperature, *k_B_* is the Boltzmann constant, and *V* is the system volume.

The benefit of this method is that it requires significantly fewer simulation cells than non-equilibrium MD. It is also suitable for perfect crystals like Si and diamond with long phonons. However, when complex potential functions are employed, this approach lacks convergence and makes it difficult to calculate the heat flux. Furthermore, when dealing with inhomogeneous systems, this approach computes a thermal conductivity average over all the systems, i.e., an interface. As a result, the detailed behavior of phonons at the interface cannot be studied. Based on these facts, it seems that this approach cannot be used to investigate the thermal properties of 2D nanomaterials containing impurities and interfaces.

### 4.7. Atomistic Green’s Functions

Since it is evident, phonons are wave-like particles. Wave effects on a discrete atomic lattice can be accurately modeled using Atomistic Green’s Functions (AGF). Initially, this method was introduced to deal with quantum electron transport in nanostructures [61,66,67,68,69,70,71]. The approach can be applied to a variety of nanostructures by making a few careful substitutions [72,73,74,75]. It is particularly suitable for low-dimensional heterostructures such as Si/Ge [76], graphene/h-BN [77], MoS_2_/metal [78] interfaces, and others [79].

## 5. Experimental Measurement

Due to the difficulty of extracting precise temperature gradients and heat fluxes, measuring the thermal conductivity of 2D materials is challenging. These nanostructures cannot be measured with traditional tools for temperature and heat flux measurements since most nanostructures are orders of magnitude smaller than the finest thermocouples. Therefore, a variety of optical and electrical tools have been utilized to measure the thermal properties of 2D materials.

There are two experimental method types in the micro/nano-scale thermal conductivity measurements: steady-state measurement (i.e., suspended thermal bridge method, Raman method, etc.) and transient measurement (i.e., 3ω method, time-domain thermal reflection technique, shock optical pulse thermal measurement method, etc.). Some 2D measurement producers are briefly discussed in the following sections.

### 5.1. Suspended Thermal Bridge Method

**Figure 5:**
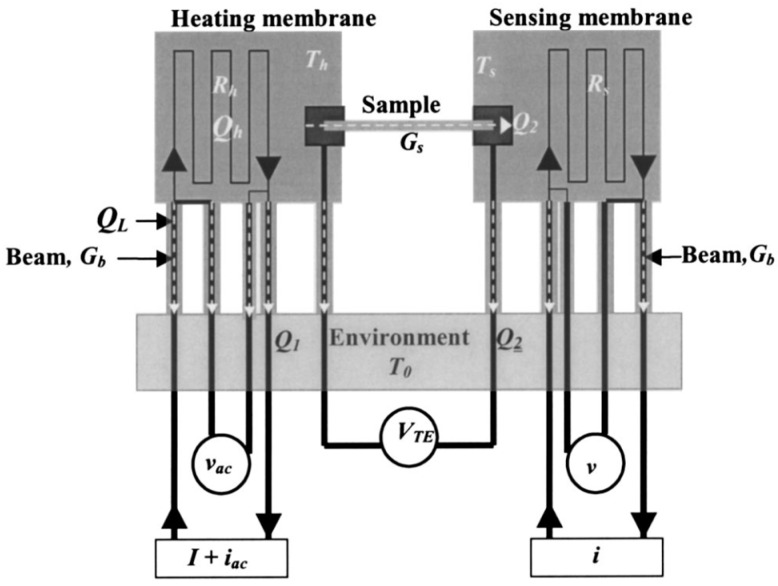
Thermal bridge method [84]. Reprinted with permission from ref. [84]. American Society of Mechanical Engineers ASME.

The invention of the suspended thermal bridge method benefits from the advancement of micro/nano processing technology development. For the first time, the suspended thermal bridge method was used in micro/nano-scale thermal conductivity measurements in 2001. The sample measured in this experiment was a single root of multi-walled carbon nanotubes [80]. Previously, traditional methods could only measure the overall thermal conductivity of a bundle of nanowires. The phonon scattering between nanowires (or nanotubes) makes it impossible to accurately determine the thermal conductivity of a single sample [81]. The suspended thermal bridge method is more useful for the study of low-dimensional thermoelectric materials [82,83]. A thermal bridge microdevice is made of two suspended silicon nitride membranes (SiN*_x_*) that are patterned with thin metal lines (Pt resistors). Figure 5 illustrates how the resistors are electrically connected to contact pads via four Pt leads and used as microheaters and thermometers, providing Joule heating and four-probe resistance measurements, respectively. The heat transfer in the suspended sample is extracted by considering the generated Joule heating on the heated membrane and the temperature change on the sensing membrane while the sample is held between the two membranes and bonded to Pt electrodes. As a result of the high accuracy of Pt thermometers and direct temperature calibration, this method can provide a high temperature resolution of ~0.05 K in a range from 4 to 400 K [80,84]. The experimentally measured thermal conductance G and thermal conductivity *k* are calculated from the equations $G=1/R_{tot}$ and $K=L/\left(AR_{tot}\right)$, respectively. Here, $R_{tot}$ is the total measured thermal resistance, *L* is known as the length of the sample, and *A* is the cross-sectional area of the sample. As mentioned, $R_{tot}$ is the total thermal resistance of the entire system, including the thermal resistance of the suspended sample, the thermal resistance contribution from the membrane-connected parts of the sample, the internal thermal resistances of the two membranes, and the additional thermal resistance contribution from the part of the membranes which are linked with the heaters/thermometers.

In recent years, there has been a massive demand for measurement of the thermal conductivity of low thermal conductivity micro/nano-scale materials. Therefore, the Wheatstone bridge method [85] and the comparator method [86] were developed to improve the measurement. Xu Xiangfan et al. [86] used the comparator method to measure the thermal conductivity of a single polyimide nanofiber. In this experiment, the thermal conductivity of the sample is about $1.0\times 10^{-10}$ W/K, which is an order of magnitude lower than the lower limit that can be measured by the ordinary thermal bridge method. It can be seen that the use of this method greatly broadens the application range of the thermal bridge method. Zheng et al. [87] used AC heating to eliminate white noise, which can further increase the measurement accuracy to about 0.25 W/K.

In spite of this, some technical challenges still need to be considered. In order to measure $R_{tot}$ accurately, it is necessary to account for the thermal contact resistance components that unavoidably contribute to them. The first component that needs to be mentioned is thermal contact resistance ($R_{c,f}$) between the two ends of the suspended sample and the SiN*_x_* membranes [88,89]. Various studies have shown the need for a fin resistance model to estimate this resistance [90,91]. The thermal contact resistance between the sample−membrane interface and the thermometer $R_{c,m}$ is another component of $R_{tot}$, as it results from a non-uniform temperature distribution on the heating membrane. The $R_{c,m}$ factor can be ignored if the membrane has a uniform temperature distribution, i.e., when the thermal resistance of the suspended sample is greater than the internal thermal resistance of the membrane. A high thermal conductivity material, such as graphene or carbon nanotubes, however, is not the case. By re-analyzing heat transport results in CVD single-layer graphene samples, Jo et al. concluded that these extrinsic thermal contact resistances contribute up to 20% of the measured thermal resistance [91]. Recently, several studies found that resistance line thermometers can be employed as a replacement for serpentine Pt thermometers to reduce the size of the temperature measurement resistance (between heater/sensor and contact point) [92,93]. It has been determined that by employing numerical heat conduction calculations, the contribution of $R_{c,m}$ decreases to about 30–40% compared to the values that correspond to the serpentine resistance thermometer [91]. The device fabrication and sample transfer are also time-consuming and complex with this technique. In most cases, exfoliated 2D materials are transferred to the thermal bridge structure using a dry transfer method, causing polymer residues, defects, and rough edges on the sample surface that greatly affect the measured total thermal resistance [32,94]. Within the temperature range of 4 to 400 K, the suspended thermal bridge method can be applied. An advanced method based on the tunnel current in a metal−insulator−superconductor junction has been proposed for sub-Kelvin measurements [95]. This allows measurements to be made down to 1 m·K

Additionally, various materials, such as nanofilms [88,89] and 2D materials, including graphene [91,96,97,98,99], boron nitride [100], and TMDC materials [101,102], have also been measured using the thermal bridge method.

### 5.2. Electron Beam Self-Heating Method

**Figure 6:**
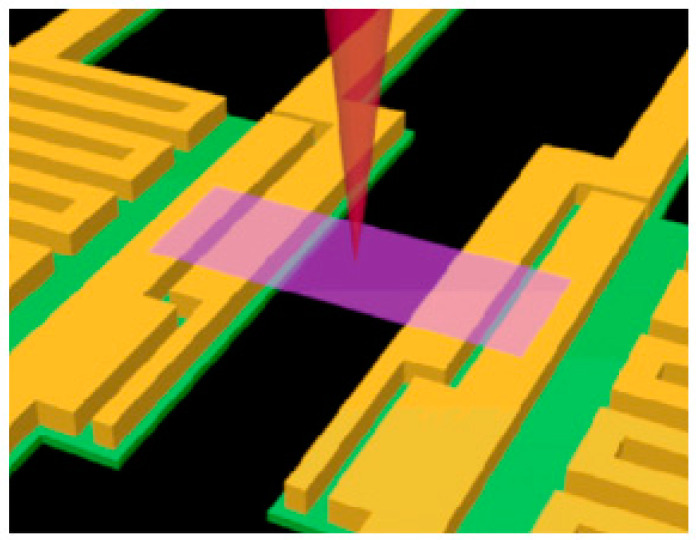
Electron beam self-heating method [104]. Reprinted with permission from ref. [104]. Elsevier and Copyright Clearance Center.

In the above-mentioned suspended thermal bridge method, the thermal contact resistance between the sample and the suspended platform is one of the main faults of this process. Although there are already some methods to improve it, the effect of this defect cannot be eliminated from the experimental principle. Researchers at Li Baowen’s lab developed the electron beam self-heating method based on the suspended thermal bridge method in 2010 [103,104]. This method omits the influence of the contact thermal resistance between the sample and the suspended platform on the experimental results in principle and measures the spatial distribution of the thermal resistance of the micro/nano-scale materials. A scanning electron microscope (SEM) is used to measure the electron beam self-heating method. As demonstrated in Figure 6, heat is generated by the interaction between the high-energy electron beam in the SEM and the sample. It is possible to scan (move on) the sample continuously.

A scanning electron beam is used as a heating source, while the two suspended membranes behave as temperature sensors in the electron beam self-heating method. Hot spots emerged as a result of the electrons’ energy absorption along the length of the sample during the scanning of the focused electron. As heat is generated from the local spots, it flows to the two membranes where it increases their temperature. The thermal conductivity of the sample can be obtained by:

(4) k=A/(dR/dx)

Here, *A* is the cross-sectional area of the sample, *x* represents the distance between the membrane and the heating spot, and *R* is the calculated thermal resistance from one membrane to the heating spot.

This method has the advantage of measuring *R* by combining the diffusive thermal resistance of the suspended part ($R_{d}$) and the thermal contact resistance between the suspended sample and contact electrodes ($R_{c}$), as shown by Equation (5):

(5) $$R=R_{d}+R_{c}$$

In this equation, ($R_{d}$) and ($R_{c}$) can be calculated by solving the following equations:

(6) $$R_{d}=L/ktW$$

(7) $$RW=L/kt+R_{c}W$$

The thermal conductivity, length, thickness, and width of the suspended sample are represented by *k*, *L*, *t*, and *W*, respectively. According to the $R_{d}$ formulation, it is evident that its value decreases as t increases and *L* decreases. Also, taking the limit L/t→0 will give $R_{c}$. However, generally in laser-based methods, the spatial resolution is restricted by the heating volume within the sample rather than the spot size. It follows that the spatial resolution of this technique is dependent on the properties of the studied materials [105]. Recent studies have used electron beam self-heating to determine the thermal conductivity and thermal resistance of suspended Si and SiGe nanowires and MoS_2_ ribbons [105,106,107]. Also, this method has been employed to measure the interfacial thermal resistance between few-layer MoS_2_ and Pt electrodes [102].

Although the advantages of the electron beam self-heating technique are evident, its drawback cannot be ignored, such as the fact that it cannot apply variable temperature measurement, cannot measure materials that are sensitive to electron beams, and is susceptible to impurities on the sample surface (organic matter, etc.).

### 5.3. Raman Method

**Figure 7:**
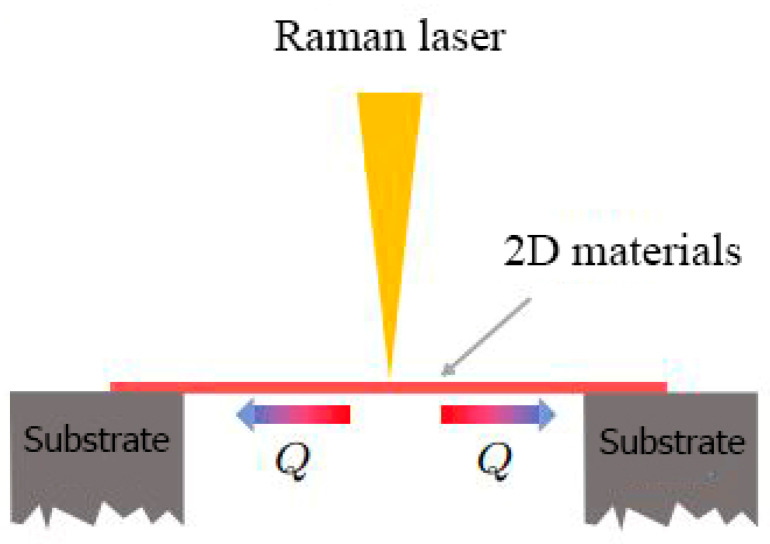
Raman spectroscopy schematic.

In 2008, the first experimental measurement of the thermal conductivity of two-dimensional material, single-layer graphene in the suspended plane was the Raman method [84]. In two-dimensional materials, the Raman method has become one of the most important experimental methods for measuring thermal conduction. Several two-dimensional materials have been measured successfully using this method, including boron nitride [108,109], black phosphorus [110], and molybdenum sulfide [56,111,112]. The Raman method can be used to measure the thermal conductivity of two-dimensional materials by taking into account the following two factors: (1) Raman lasers can be used as heat sources because 2D materials have an absorption effect on them; (2) The Raman spectrum absorption peak positions of two-dimensional materials and a certain linear relationship between temperature [110,112,113]; in this way, the surface temperature of the material can be determined by the Raman spectrum of the material. The thermal conductivity of a two-dimensional material can be calculated by combining the two principles mentioned above through the heat conduction model. A schematic of Raman spectroscopy is shown in Figure 7. Using Raman peaking shifting, the temperature is measured for the sample [30,114]. Obtaining the temperature can be achieved since the Raman peak is a linear function of the temperature. Thermal conductivity can be measured based on the absorbed power and temperature.

As an optical method, Raman studies the phonons and vibrational modes of molecular vibrations in solids. In this method, the inelastically scattered light of a monochromatic laser beam that interacts with a material is studied. An oscillating dipole moment is generated as a result of the oscillating electromagnetic field of the incident light which acts as a radiation source causing Raman scattering. Based on the nature of the chemical bonds and the crystal structure, each material or solid crystal has a characteristic set of molecular vibrations and phonons. Materials can be characterized in an elementary and structural manner using this technique. In addition, this method can be used to determine small changes in the crystal structure resulting from embedded strain, thermal expansion, sample composition, and structural disorder, impurities, and contamination, as well as pseudo-phases and deformation of the material [115,116,117,118].

With the continuous development and improvement of the Raman method, it has become one of the most accepted methods of micro/nano scale heat conduction measurement. In a recent review article, Malekpour and Balandin provide a detailed description of Raman-based techniques to measure the thermal properties of graphene and related materials [114].

There is, however, considerable uncertainty associated with this method due to the uncertainty of absorptivity. By using a method known as energy transfer state-resolved Raman (ET-Raman), the accuracy of this method can be improved in order to overcome this issue [119]. To achieve this, two steady-state lasers with different sizes are used to heat the sample and measure the temperature shifts as a result. The absorptivity term in this method will be canceled and the signal-to-noise ratio will be improved significantly with this method. In this way, the measurements made with this method are more accurate than those performed using the original optothermal Raman method.

### 5.4. Time-Domain Thermoreflectance Method

**Figure 8:**
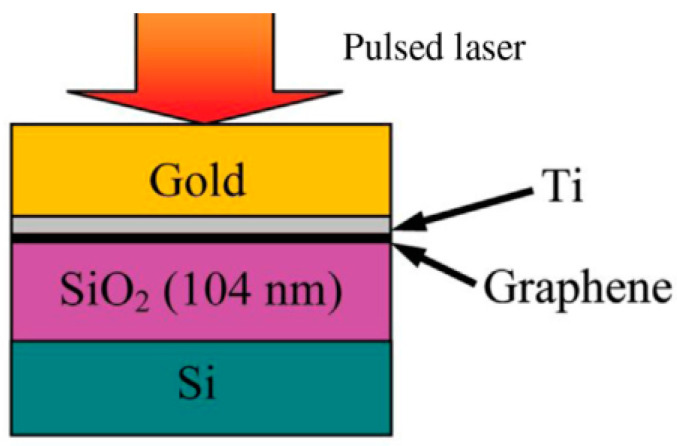
Time-domain thermoreflectance diagram (TDTR) [125]. Reprinted with permission from ref. [125]. Copyright 2003 American Chemical Society.

In 1983, Eesley applied the picosecond pulsed laser to detect the non-equilibrium heat transport process in metallic copper [120] since the time-domain thermoreflectance (TDTR) method has been formally applied to the measurement of material thermal properties. The TDTR method has been developed over a period of thirty years. It has now become one of the most widely used methods for measuring the thermal properties of materials in an unsteady state. This method is usually employed to measure the thermal conductivity and interfacial thermal resistance of materials [92,93,121]. Its basic principle is that a beam of a femtosecond pulsed laser is divided into a pump light and a probe light through a beam splitter. In this system, the pump light is used as a heat source for heating the surface of the material, and the probe light measures the change in the surface temperature of the material (the reflectivity of the material surface to the laser is related to the temperature). The displacement platform can accurately control the optical path difference between the two beams and then control the time interval between them to reach the surface of the material, resulting in a certain time delay (*t_d_*). A schematic is illustrated in Figure 8. The temperature change process is related to the thermophysical properties of the material.

The measurement system of the TDTR includes a femtosecond laser generator, beam splitter, displacement platform, electro-optic (acousto-optic) modulator, photodiode detector, lock-in amplifier, etc. [122]. Before measurement, generally, it is necessary to coat a metal film on the sample surface to be tested as the sensing layer because the reflectivity of the metal surface to the laser is approximately linear with the temperature under the condition of a small temperature rise, and the surface temperature can be calibrated more accurately through the above measurement process. The lock-in amplifier will output the in-phase signal (*V_in_*) and the inverted signal (*V_out_*) based on the modulation frequency, which contains the information of the temperature change of the sample surface, and then the in-phase signal and the inverted signal can be obtained. Finally, the thermal conductivity model is derived and the experimental data is fitted to extract the correlation of the thermal properties of the sample data. Measurements of the thermophysical properties of 2D materials, such as graphene [123], black phosphorus [55], molybdenum sulfide [92,93], and tungsten selenide [124], including thermal interface resistance between the graphene and SiO_2_ [125], have been conducted using the TDTR method.

Compared with the steady-state thermal measurement method, TDTR does not require measurement in a vacuum chamber. Secondly, it can be applied for ultra-fast thermal transport mechanism research (i.e., electro-acoustic interaction, etc.). Some 2D materials, especially single-layer and multi-layer materials, adsorb impurities or deposits on their surfaces in order to suppress acoustic phonons out of plane, so this method cannot accurately measure the intrinsic heat of these materials [126].

### 5.5. Micro-Suspended-Pad Method

The suspended-pad method, first used to measure carbon nanotubes [80] and silicon nanowires [126,127], is another method frequently used for nanostructures, nanoribbons, and 2D materials. The micro-pad devices are manufactured in batches as part of this method. A device consists of two adjacent silicon nitride membranes suspended by a silicon nitride long beam [127]. The patterned platinum heaters are manufactured on both the pads and long beams. Normally, samples are transferred using a nanomanipulator. Utilizing focused ion-beam deposition, the thermal contacts can be increased by Pt deposition, making the contacts electrically and thermally ohmic. It is, therefore, possible to ignore the thermal resistance of the junction. Various 2D materials have been measured using the suspended-pad method, such as h-BN [32], black phosphorus [128], and MoS_2_ [129]. Due to the accuracy of this method, the electrical signal can be very precisely derived. In addition, the interpretation of the data is very clear. The input current can accurately control the heat flux, and the temperature can be precisely measured via temperature-dependent electrical resistance. Thermal conductivity can therefore be accurately measured with the heat flux across the pads and the temperature on both pads (usually the uncertainty level is under 5%). A few factors limit its application, despite its effectiveness in measuring the thermal transport properties of nanostructures. First, it is heavily dependent on intricate manufacturing. There is also the possibility of defects arising from sample preparation as a second factor. This includes the polymer residues on BN samples [43] or the defects in black phosphorus [128]. Before using this method, it is important to take into account these factors.

## 6. Research Progress on 2D Thermal Conductivity

The thermal conductivity of various two-dimensional materials has been studied, and research progress on the thermal conductivity of the most regular 2D materials is summarized here.

### 6.1. Graphene

As the first 2D material successfully prepared, graphene became the favorite of scientific research once discovered. It has a series of excellent physical properties including ultra-high conductivity, ultra-high carrier mobility, etc. [16,130,131]. In terms of thermal conductivity, graphene also performs well, and its intrinsic thermal conductivity at room temperature can reach 2000–3000 W/m·K, which is the highest thermal conductivity material found so far. In 2008, for the first time, Balandin et al. [40] measured the thermal conductivity of suspended single-layer graphene at room temperature using the Raman method and graphite bulk materials reaching 4840–5300 W/m·K; however, upon further study by scientists, it was discovered that the experiment may have had an excessive estimate of the Raman laser absorption power of graphene, resulting in a result 4–6 times larger [30,132]. In 2010, Wei et al. [133] used the same method to measure the thermal conductivity of suspended single-layer graphene and, by employing a laser power meter, measured the Raman laser absorption rate of graphene simultaneously.

The results illustrate that the thermal conductivity of single-layer graphene grown by chemical vapor deposition (CVD) is around 2500–3100 W/m·K (*T* = 350 K) and 1200–1400 W/m·K (*T* = 500 K). It has also been found that the shape, size, and measurement environment of the suspended part of the graphene will affect the final result. In addition to the dispute about the laser absorption rate, it caused the difference between different experimental results. Another major reason for the large difference is that the preparation methods of graphene are different (mechanical exfoliation or chemical vapor deposition), resulting in certain differences in its quality (impurities, grain boundaries, organic residues, etc.). These factors will affect the phonon and generate additional scattering. Table 1 lists the thermal conductivity of suspended single-layer/multi-layer graphene measured by different experimental methods, including the Raman method.

In order to understand graphene’s ultra-high thermal conductivity, it is important to know how different phonon modes contribute to it. In graphene, heat is mainly transmitted through acoustic phonons, and its acoustic phonon modes include in-plane acoustic longitudinal wave (LA), in-plane acoustic transverse wave (TA), and out-of-plane acoustic shear wave (ZA). Nika et al. [140] believe that the heat transport process in single-layer graphene is almost entirely carried by the LA/TA phonon mode, while the contribution of the ZA phonon mode to the thermal conductivity is negligible. However, according to Lindsay et al. [52,53], the ZA phonon mode has a relatively high density of low-frequency phonons, so its contribution to the thermal conductivity will be relatively large, and it is predicted that the contribution of the ZA phonon mode to the thermal conductivity of single-layer graphene can reach about 70% at room temperature. In addition to ultra-high thermal conductivity, graphene is also an excellent platform for studying phonon ballistic transport. Because the mean free path of phonons in graphene is very long, it can reach the order of micrometers at room temperature, so the (quasi) ballistic transport properties of phonons can be studied by adjusting the length and width of the graphene band [141]. In 2010, Xu Xiangfan et al. used the suspended thermal bridge technique to measure the monolayer for the first time. The thermal conductivity of CVD graphene nanoribbons (width of about 3 µm, length of about 500 nm) is found to have a certain exponential relationship between thermal conductivity and temperature in the low-temperature region (T < 140 K), which can be explained by the thermal transport in single-layer suspended graphene, mainly depends on the ZA phonon mode, and experimental evidence of phonon quasi-ballistic transport has been found [97,138]. Although Petters et al. [98] object to the low thermal conductivity (about 225 W/m·K) measured in the above experiment, it is believed that the observed *k*~1.53 ± 0.18 is not from ZA phonons but due to impurities on the surface of the sample.

The thermal conductivity theory discussed above is calculated based on the three-phonon scattering model. Four-phonon scattering is often directly ignored because it is a type of phonon scattering behavior that only appears in high-temperature regions [142]. However, recent studies found that even at room temperature, the four-phonon scattering process caused by a large number of low-energy ZA mode phonons in single-layer graphene cannot be ignored. Therefore, the thermal conductivity of single-layer graphene, calculated using only the three-phonon scattering model, may be relatively high [142,143]. By solving the Boltzmann equation and introducing four-phonon scattering, Feng et al. [142] found that the thermal conductivity of single-layer graphene at room temperature is only 810 W/m·K; this value is much lower than the calculation result including only three-phonon scattering (about 3383 W/m·K), and the result shows that under the influence of four-phonon scattering, the contribution of the ZA phonon mode to the thermal conductivity is only 31%. The atomic force constant used in this work to calculate the thermal conductivity of single-layer graphene at room temperature is 0 K. Subsequently, Gu Xiaokun et al. [143] corrected the above results, and the result value obtained was slightly larger than that in the above literature, further confirming the severe impact of four-phonon scattering on the thermal conductivity of single-layer graphene at room temperature. It can be seen that the thermal conductivity of single-layer-suspended graphene is still inconclusive. There is a certain controversy about this problem, whether in theory or in experiments. In practical applications, graphene is more likely to be attached to a certain substrate.

Therefore, in addition to floating graphene, the in-plane heat conduction of graphene on substrate performance is also necessary to study. Seol et al. [52] measured the in-plane thermal conductivity of a single layer of graphene on a silicon oxide substrate (about 600 W/m·K, 300 K). When the graphene is attached to the substrate, the ZA phonon mode will be suppressed, so its in-plane thermal conductivity is lower than that of suspended graphene. As shown in the above theoretical calculation results, the contribution of the ZA phonon to the thermal conductivity can reach about 70% at room temperature, but when there is a lining at the end, the contribution of the ZA phonon mode will be severely suppressed or even disappear, and the thermal conductivity value will be reduced from ~3000 W/m·K to ~600 W/m·K. This experiment effectively verifies that the graphene ZA phonons make a significant contribution to its thermal conductivity. In addition to silicon oxide, silicon nitride is also a common substrate material. Thong et al. [103] measured the in-plane thermal conductivity of multi-layer graphene on a silicon nitride substrate. The value is ~150–1250 W/m·K (room temperature). In order to further verify the contribution of ZA phonons to thermal conductivity, Wang et al. [99] deposited gold atoms on the surface of three-layer suspended graphene. It was found that its thermal conductivity decreased from about 1500 W/m·K to about 270 W/m·K (a decrease of 82%) and Seol et al. came to the same conclusion. Jang et al. [144] studied the heat transport properties of the SiO_2_−graphene−SiO_2_ sandwich structure and found that the thermal conductivity will be further reduced, especially the single-layer sandwich graphene structure. Iits room temperature thermal conductivity is far below 160 W/m·K (the specific value in this article is too low to be measured and only an upper limit is given), indicating that the substrate has a very significant inhibitory effect on the thermal conductivity of graphene.

### 6.2. Boron Nitride

Due to the large bandgap and the very smooth surface, boron nitride (h-BN) is an ideal type of dielectric material. At the same time, the thermal conductivity of boron nitride bulk materials (about 400 W/m·K, room temperature) is very close to copper, and its mass is much lower than copper under the same volume, so it has broad application prospects in terms of the heat dissipation of electronic devices [145,146]. Boron nitride is called white graphene. The crystal structure is similar to graphene. Nitrogen atoms and boron atoms in the plane are interlaced to form a honeycomb structure, and the layers are combined with each other by van der Waals forces. It is one of the two-dimensional materials discovered earlier [147]. The physical properties of boron nitride and graphene also have certain similarities. Lindsay et al. [148] predicted the room temperature in-plane thermal conductivity of single-layer boron nitride through theoretical research by solving the Boltzmann equation. The rate is 600 W/m·K, which is higher than that of the boron nitride bulk material. At the same time, it is also found that the contribution of the out-of-plane ZA phonon mode to the thermal conductivity can reach ~60%. In 2013, Jo et al. [32] adopted a microbridge resistance thermometer method to measure the in-plane thermal conductivity of multi-layer boron nitride (250 W/m·K, five layers; 360 W/m·K, 11 layers; *T* = 300 K). They believe that the reason why the measured data is lower than the theoretical prediction and even lower than the thermal conductivity of the boron nitride bulk material is mainly due to a large amount of organic residue on the surface of the boron nitride during the experiment, which causes serious phonon scattering. In the subsequent experiments of the thermal conductivity of the multi-layer boron nitride floating plane, it was not observed that the thermal conductivity exceeds the bulk material, and the quality of the sample is the key factor [108,113,149]. Wang et al. [100] improved the sample transfer method (PDMS-assisted dry transfer), which greatly reduced the organic residues on the surface of the boron nitride film. At the same time, they used high-quality bulk materials to mechanically peel off the multi-layer boron nitride and for the first time, measured the thermal conductivity of the suspended double-layer boron nitride as being greater than that of the bulk material, reaching 460–625 W/m·K at room temperature. Subsequently, Cai et al. [109] used the Raman method to measure the thermal conductivity of single-layer/double-layer and three-layer floating boron nitride near room temperature. The thermal conductivity value decreases as the thickness increases, but it is still higher than bulk boron nitride. The thermal conductivity of single-layer boron nitride reaches ~751 W/m·K. The highest value of the thermal conductivity of single-layer/multi-layer boron nitride was obtained in previous experiments. Table 2 lists the in-plane thermal conductivity of floating boron nitride measured in different documents. According to the data in the table, the thermal conductivity of multi-layer boron nitride prepared by the mechanical exfoliation method is generally higher than that prepared by the CVD method. The reason is that the chemical vapor deposition method often introduces more in the sample. However, the mechanical exfoliation method performs better at controlling the sample quality.

### 6.3. Molybdenum Sulfide and Other Transition Metal Sulfides

**Figure 9:**
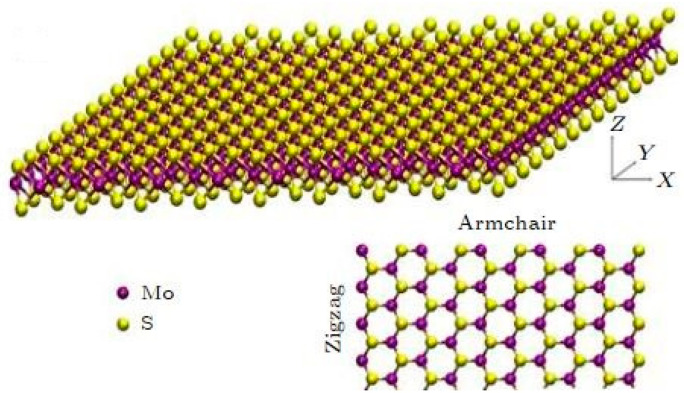
Molybdenum sulfide [151]. Reprinted with permission from ref. [10]. AIP Publishing and Copyright Clearance Center.

Transition metal sulfides (MX_2_, where M is transition metal elements such as Mo, W, Ti, and X represents chalcogen elements, including S, Se, and Te) are a very important group of two-dimensional materials and their crystal structure is a “sandwich”-like layered structure [150]. Unlike single-layer graphene, single-layer boron nitride, and other two-dimensional materials that only contain one atomic layer, a single-layer transition metal sulfide contains three atomic layers (the transition metal atomic layer is sulfurized). The atomic layer of group elements is “sandwiched” in the middle, as shown in Figure 9.

Molybdenum sulfide is the most widely studied transition metal sulfide. Because of its controllable bandgap and excellent electrical properties, it can also exist stably in the air. It is considered a material for the next generation of microelectronic devices with great potential. Optics, thermoelectrics, and other fields also have certain application prospects [151,152,153]. Sahoo et al. [112] used the Raman method to measure the in-plane thermal conductivity of 11 layers of molybdenum sulfide (about 52 W/m·K, room temperature). Later, Yan et al. [56] and Jo et al. [129] measured the in-plane thermal conductivity of single-layer and multi-layer molybdenum sulfide at room temperature and the values were 35–52 W/m·K. However, Zhang et al. [154] also used the Raman method to measure the in-plane thermal conductivity of single-layer/double-layer molybdenum sulfide, and the result (77–84 W/m·K) was much larger than the previous experimental data. It is because the critical data, such as the relationship between the Raman peak frequency change and temperature of molybdenum sulfide, the absorption power of the Raman laser, and the contact thermal resistance obtained in the experiment are quite different from the previous literature. Aiyiti et al. [104] used the electron beam self-heating method to measure the in-plane thermal conductivity of the multi-layer molybdenum sulfide. This is the first time this method has been applied to the experimental measurement of the thermal conductivity of two-dimensional materials. The experimental results also confirm the feasibility of this method. The results of the experimental measurements of the in-plane thermal conductivity of molybdenum sulfide are summarized in Table 3.

Compared with graphene and boron nitride, the crystal structure of molybdenum sulfide has certain differences, so its thermal conductivity properties will also be different, mainly as follows: First, the thermal conductivity of single-layer molybdenum sulfide is higher than that of single-layer graphene and single-layer nitrogen. The boron sulfide is 1-2 orders of magnitude lower. Wei et al. [35] found through theoretical calculations that the low thermal conductivity of single-layer molybdenum sulfide is due to the lower phonon group velocity and the larger Grüneisen constant. As a result, the mean free path of phonons is only 14.6 nm. Secondly, in single-layer graphene and single-layer boron nitride, the contribution of the out-of-plane ZA phonon mode to the thermal conductivity is more than 50%, but in a single layer of molybdenum sulfide, the contribution of the in-plane phonon mode to the thermal conductivity exceeds the out-of-plane phonon mode. Finally, unlike graphene and boron nitride, single-/multi-layer molybdenum sulfide has been experimentally measured. The in-plane thermal conductivity is lower than the in-plane thermal conductivity of molybdenum sulfide block (85–110 W/m·K, room temperature). This phenomenon is inconsistent with the theoretical prediction. Gu et al. [57] predicted that the room temperature in-plane thermal conductivity of a single layer of molybdenum sulfide could reach 138 W/m·K. The reason may be that the quality of molybdenum sulfide in these experiments has not reached a good condition, or the deeper reasons need to be further studied. In addition to the in-plane thermal conductivity, the inter-plane thermal conductivity of molybdenum sulfide is also one of the issues worthy of study, but there are relatively few studies in this direction. Initially, Muratore et al. [159] and Cahill et al. [160] measured the room temperature interfacial thermal conductivity of bulk molybdenum sulfide, which was only 2–3 W/m·K. However, Jiang et al. [93] showed a higher numerical result (about 4.75 W/m·K) experimentally, and the result is closer to the theoretical prediction. The difference between the above experimental results is in the follow-up Sood et al. [92] explained in the experiment. They measured the room-temperature inter-plane thermal conductivity of different thicknesses of molybdenum sulfide by the TDTR method, and the results showed that the room-temperature inter-plane thermal conductivity of samples with a thickness of 240 nm and 20 nm were 2.0 ± 0.3 W/m·K, 0.9 ± 0.2 W/m·K. Through comparison with the above experimental data, it is found that as the thickness increases, the inter-face thermal conductivity of molybdenum sulfide increases, and when the thickness reaches about 1 µm, the inter-face thermal conductivity value gradually approaches the saturation threshold (about 5 W/m·K). Theoretical calculation results show that the mean free path of phonons between the molybdenum sulfide surfaces far exceeds the previously estimated value (1.5–4 nm), and more than 80% of the heat transport is contributed by phonons with a mean free path of 10–500 nm.

With the continuous development of the preparation technology of two-dimensional materials, more and more multi-layer transition metal sulfides have been discovered, so their thermal conductivity is gradually being studied. Table 4 lists other transitions in different literatures except for molybdenum sulfide. Experimental measurement of the thermal conductivity of metal sulfides in the suspended plane. From the data in the table, it can be seen that although the crystal structures of these materials are very similar, their thermal conductivity properties are significantly different.

### 6.4. Black Phosphorus, Black Arsenic

Due to the advantages of the controllable bandgap and relatively high switching, black phosphorous (BP) is one of the first materials for the next generation of microelectronic devices to be studied [168,169,170]. However, initially, researchers were interested in the thermal conductivity of black phosphorous. This is mainly because of its in-plane anisotropic “Great Wall”-like structure [128], which may lead to the anisotropy of thermal conductivity [171]. It is worth noting that the black phosphorous pole is easy to oxidize, so in experiments with black phosphorus, the exposure time of the sample in the air needs to be strictly controlled. Qin et al. [172] predicted the room temperature surface of the single-layer black phosphorus along the Zigzag (ZZ) direction and the (Armchair) AC direction through theoretical research. The internal thermal conductivity ratio can reach 30.15 W/m·K in the ZZ direction and 13.65 W/m·K in the AC direction, and due to its “Great Wall”-like structure, the out-of-plane phonon mode has a positive effect on the thermal conductivity. The contribution of efficiency is very low (about 5%). Lou et al. [110] experimentally measured the in-plane thermal conductivity of the multi-layer black phosphorus with different thicknesses at room temperature and the smallest thickness was ~10 nm. The thermal conductivity in the ZZ direction is 20 W/m·K, while the thermal conductivity in the AC direction is only ~10 W/m·K, which confirms the above theoretical prediction. As the same main group element of phosphorus, arsenic, i.e., black arsenic (Bas), has a crystal structure similar to black phosphorus and also has a significant in-plane thermal conductivity anisotropy effect. Chen et al. [173] first experimentally measured the in-plane thermal conductivity of black arsenic with a thickness of 124 nm along with the ZZ and AC directions (5 W/m·K, ZZ direction; 3 W/m·K, AC direction, 350 K). In subsequent experiments, researchers measured the in-plane thermal conductivity of multi-layer black phosphorus with different thicknesses using the Raman method, thermal bridge method, etc. They found the anisotropy of in-plane thermal conductivity. In these experiments, the thickness of the multi-layer black phosphorus was above 10 nm. This is because the chemical properties of black phosphorus are too active and it is challenging to prepare single-layer black phosphorus in heat conduction experiments. Therefore, the heat conduction properties of single-layer or few-layer black phosphorus need to be further studied.

### 6.5. Telluride

Bulk tellurium (Te) is a new and high-quality thermoelectric material [174]. At the same time, due to its two-dimensional structure, telluride can be used as an effective means to further improve its thermoelectric properties [175]. In the bulk material, the tellurium atom is combined with a neighboring atom through a covalent bond and extends in a spiral shape. Adjacent spiral chains are then combined by van der Waals forces [176], so bulk tellurium belongs to a quasi-one-dimensional chain structure. However, the theory predicts that the structure of monolayer telluride is different from that of bulk tellurium. There are three possible crystal structures (α-Te, β-Te, γ-Te) [177]. Gao et al. [178,179], through first principles calculations, studied the thermal conductivity and thermoelectric properties of single-layer telluride with different structures, and they have been found to have low thermal conductivity and excellent thermoelectric properties. But only b-type telluride has been synthesized in experiments and the synthesis conditions are relatively harsh, so there is no experimental study on the physical properties of monolayer telluride [180,181]. Wang et al. developed a liquid-phase synthesis method [182], which can be used to prepare multi-layer telluride with the same bulk structure in large quantities. In further research, the research group and its collaborators used the Raman method to measure the room temperature suspended thermal conductivity of telluride with a thickness of 35 nm in the intra-chain direction (about 1.5 W/m·K) and compared with the bulk material (about 3 W/m·K, 300 K), there is a certain degree of reduction. They believe that the main reason for this is that the surface of the multi-layer telluride will interact with the phonons. Because the structure of this type of telluride is the same as that of the bulk material, its in-plane thermal conductivity will be different along the in-chain direction and the inter-chain direction.

### 6.6. Silicene

After the discovery of graphene, as an element of the same family of carbon, silicene was naturally noticed. However, it was not until 2012 that silicene was synthesized, and the growth conditions were too harsh [183,184]. Therefore, the thermal conductivity of silicene is still in theoretical research and there has been no progress in experimental research. In addition to the intrinsic thermal conductivity, theoretically, the use of stress, electric fields, defects, isotopes, and other methods to control the thermal conductivity of silicene has been studied in theory, which will provide guidance for future experimental research of silicene.

### 6.7. Other 2D Materials

With the continuous development of nanomaterial preparation technology and computational simulation technology, the family of two-dimensional materials is becoming larger and larger. Not only can a new type of two-dimensional material be synthesized experimentally, but it is also possible to predict some unknown two-dimensional materials through theoretical simulation. In addition to the above-mentioned materials, there are many other two-dimensional materials whose thermal conductivity properties have also been studied. In terms of experiments, researchers have used different methods to measure multi-layer bismuth telluride [185], multi-layer indium selenide [186], and the in-plane thermal conductivity of two-dimensional materials such as multi-layer tin sulfide [187], zirconium telluride [83], and multi-layer bismuth selenium oxide [188]. Thermal conductivity and related properties of two-dimensional materials such as gallium nitride [189], boronene [190], single-layer carbon nitride [191], and single-layer nitrogen boron carbide [192] have also been studied.

## 7. Summary

To sum up, this article uses the thermal conductivity of 2D materials as a research platform to discuss the most basic physical issues of heat conduction at the micro/nano scale. Compared with ten years ago, we have a certain understanding of the thermal conduction mechanism of two-dimensional materials. However, there is a long way to go, and there are still many problems in the study of the heat conduction of two-dimensional materials, but this can also point out the need for further research related to the heat conduction of two-dimensional materials.

Here are some issues that need to be considered: (1) There is no rigorous analytical solution for abnormal heat conduction in two-dimensional materials. Existing models for abnormal heat conduction are limited to two-dimensional lattices; (2) What is the thermal conductivity of graphene? There are significant differences between different experiments, and the calculation results of three-phonon scattering and four-phonon scattering are not self-consistent. With the current experimental measurement technology, the measurement of the intrinsic thermal conductivity of single-layer floating graphene seems to be an impossible task; (3) In the current theoretical framework, four-phonon scattering is often directly ignored because it is a type of phonon scattering behavior that only gradually appears in high-temperature regions. However, some theoretical studies have found that even at room temperature, the four-phonon dispersion process is caused by a large number of low-energy ZA mode phonons in single-layer graphene, and this cannot be ignored. Therefore, it is necessary to re-examine the thermal conduction behavior of two-dimensional materials with four-phonon scattering theory; (4) Although theoretical work has shown that even when the scale of the sample reaches the order of millimeters or even centimeters, there is still a scale effect of thermal conductivity; (5) Thermal conductivity measurements, such as the Raman method and thermal bridge method, inevitably have contact thermal resistance problems, which will greatly affect the experimental results. Although some research groups use the dual Raman laser method and electronic beam self-heating method to eliminate the influence of contact thermal resistance, harsh experimental conditions and expensive experimental equipment make it impossible for most research groups to carry out related experiments; (6) The commonly used interfacial thermal conductivity measurements, such as the TDTR and 3w methods, can provide micron-scale spatial resolution, but they can only be used to measure the interfacial thermal resistance of thin-film materials, and their in-plane spatial resolution is also limited to the spot size of the heating laser (usually ~micrometers). Therefore, it is necessary to develop a new measurement method, which needs a spatial resolution that can reach the nanometer scale and detect the interfacial thermal resistance information of two-dimensional materials.

## Figures and Tables

**Table 1:** Experimental detail of thermal conductivity in suspended single-/few-layer graphene from different studies.

Preparation Method	Graphene Layers	Thermal Conductivity k/W (m·K)^−1^
Raman Method		
Mechanical exfoliation [30]	1	~4840–5300 (Room temperature)
Mechanical exfoliation [132]	1	~3080–5150 (Room temperature)
CVD [133]	1	~2500 +1100/−1050 (*T* = 350 K)
CVD [133]	1	~1400 +500/−480 (*T* = 500 K)
CVD [134]	1	~2600–3100 (*T* = 350 K)
Mechanical exfoliation [135]	1	~630 (*T* = 660 K)
Mechanical exfoliation [136]	1	~1800 (*T* = 325 K)
Mechanical exfoliation [136]	1	~710 (*T* = 500 K)
CVD [137]	1	~850–1100 (*T* = 303–644 K)
Mechanical exfoliation [137]	1	~1500 (*T* = 330–445 K)
Mechanical exfoliation [137]	2	~970 (*T* = 303–630 K)
Suspended thermal bridge method		
CVD [138]	1	~190 (*T* = 280 K, *L* = 0.5 µm)
CVD [98]	2	~560–620 (Room temperature, *L* = 5 µm)
CVD [97]	1	~1689–1831 (*T* = 300 K, *L* = 9 µm)
Scanning thermal microscopy (SThM)		
CVD [139]	1	~2100–2430 (*T* = 335 K)

**Table 2:** Experimental results of thermal conductivity of suspended single-/few-layer h-BN in different kinds of studies.

Number of Boron Nitride Film Layers	Preparation Method	Measurement Methods	Thermal Conductivity (Room Temperature/300 K) *k/*(W(m·K)^–1^)
5	Mechanical exfoliation [32]	Microbridge thermometer	~250
11	Mechanical exfoliation [32]	Microbridge thermometer	~360
9	CVD [113]	Raman method	~227–280
2.1 nm	CVD [108]	Raman method	~223
10 nm/20 nm	CVD [149]	Steady/transient state	~100
2	Mechanical exfoliation [100]	Thermal bridge method	~484 +141/−24
4	Mechanical exfoliation [44]	Thermal bridge method	~286
1	Mechanical exfoliation [109]	Raman method	751 ± 340
2	Mechanical exfoliation [109]	Raman method	646 ± 242
3	Mechanical exfoliation [109]	Raman method	602 ± 247

**Table 3:** Experimental results of thermal conductivity of single-/few-layer MoS_2_ in different kinds of studies.

Preparation Method	Molybdenum Sulfide Film Layers	Measurement Methods	Thermal Conductivity (Room Temperature/300 K) *k/*(W(m·K)^–1^)
CVD [112]	11	Raman method	~52
Mechanical exfoliation [56]	1	Raman method	34.5 ± 4
Mechanical exfoliation [129]	4	Thermal bridge method	~44–45
Mechanical exfoliation [129]	7	Thermal bridge method	~48–52
Mechanical exfoliation [154]	1	Raman method	84 ± 17
Mechanical exfoliation [154]	2	Raman method	77 ± 25
Mechanical exfoliation [104]	4	Electron beam self-heating	34 ± 6
Mechanical exfoliation [104]	5	Electron beam self-heating	30 ± 3
CVD [155]	1	Raman method	13.3 ± 1.4
CVD [155]	2	Raman method	15.6 ± 1.5
CVD [156]	1	Thermal bridge method	~21–24
CVD [111]	1	Raman method	60.3 ± 5.2
CVD [111]	2	Raman method	38.4 ± 3.1
CVD [111]	3	Raman method	44.8 ± 5.9
CVD [111]	4	Raman method	36.9 ± 4.9
Mechanical exfoliation [157]	1	Raman method	~62.2
Mechanical exfoliation [158]	4	Raman method	60.3 ± 5

**Table 4:** Experimental detail of thermal conductivity of single-/few-layer transition metal dichalcogenides in different literatures.

Preparation Method	Number of Film Layers	Measurement Methods	Thermal Conductivity (Room Temperature/300 K) *k/*(W(m·K)^−1^)
Molybdenum Selenide (MoSe_2_)			
Mechanical exfoliation [154]	1	Raman method	59 ± 18
Mechanical exfoliation [154]	2	Raman method	42 ± 13
Mechanical exfoliation [161]	45 nm	Raman method	11.1 ± 0.4
Mechanical exfoliation [161]	140 nm	Raman method	20.3 ± 0.9
Mechanical exfoliation [162]	5 nm	Raman method	6.2 ± 0.9
Mechanical exfoliation [162]	36 nm	Raman method	10.8 ± 1.7
Tantalum Selenide (TaSe_2_)			
Mechanical exfoliation [163]	45 nm	Raman method	~9
Mechanical exfoliation [163]	55 nm	Raman method	~11
Tungsten Sulfide (WS_2_)			
CVD [36]	1	Raman method	~32
CVD [36]	2	Raman method	~53
CVD [111]	1	Raman method	74.8 ± 17.2
Tungsten Selenide (Wse_2_)			
CVD [111]	1	Raman method	66 ± 20.9
Mechanical exfoliation [164]	1	Raman method	36 ± 12
Tungsten Telluride (Wte_2_)			
Mechanical exfoliation [165]	220 nm	TDTR	~2
Mechanical exfoliation [166]	11.2 nm	Raman method	~0.639–0.743
Rhenium sulfide (ReS_2_)			
Mechanical exfoliation [167]	150 nm	TDTR	~50–70

## Data Availability

Not applicable.
